# The international medical elective in Nepal: perspectives from local patients, host physicians and visiting students

**DOI:** 10.5116/ijme.5dc3.1e92

**Published:** 2019-11-22

**Authors:** Devon McMahon, Rajeev Shrestha, Biraj Karmacharya, Shrinkhala Shrestha, Rajendra Koju

**Affiliations:** 1 Harvard Medical School, Boston, MA, USA; 2 Department of Pharmacology, Kathmandu University School of Medical Sciences, Dhulikhel, Nepal; 3 Department of Community Programs, Dhulikhel Hospital-Kathmandu University Hospital, Dhulikhel, Nepal; 4 Department of Cardiology, Dhulikhel Hospital-Kathmandu University Hospital, Dhulikhel, Nepal

**Keywords:** Global health, international educational exchange, international health elective, international medical elective, medical education, medical elective, medical student education, medical students, Nepal

## Abstract

**Objectives:**

To understand the impact of the international medical elective (IME) on Nepali patients and physicians alongside visiting European and American medical students.

**Methods:**

At a hospital in Nepal, semi-structured interviews were conducted with 15 patients and 15 physicians about positive and negative experiences with visiting medical students. Likert scale surveys about knowledge of Nepal, clinical competencies, and post-elective feedback were administered to 56 visiting medical students before and after their elective. Interviews were coded using conventional content analysis and surveys were analyzed using descriptive statistics and paired t-tests.

**Results:**

Emergent positive themes from interviews were that visiting students enhanced the reputation of the hospital, afforded financial benefits, improved international collaboration, and increased knowledge, culture and language exchange. However, negative themes were the language barrier and time expended to orient students. Before vs. after the elective, visiting students had increased knowledge of Nepal’s healthcare system (M=1.9, SD=0.6 vs. M=3.2, SD=0.6, t_(55)_=-10.22, p<.001), ability to communicate with health professionals from different backgrounds (M=3.3, SD=0.7 vs. M=3.6, SD=0.7, t_(55)_=-3.11, p=0.003) and practice in resource constrained environments (M=2.4, SD=0.9 vs. M=2.8, SD=0.9, t_(55)_=-2.42, p=0.02). However, students had no change in history (M=4.0, SD=0.7 vs. M=3.9, SD=0.7, t_(55)_=0.84, p=0.40), physical exam (M=3.9, SD=0.6 vs. M=3.9, SD= 0.7, t_(55)_=0.22, p=0.82) or diagnostic (M=3.5, SD=0.7 vs. M=3.4, SD=0.8, t_(55)_=1.52, p=0.14) abilities.

**Conclusions:**

This study demonstrated a variety of benefits and harms of the IME. To improve the IME experience, medical educators should emphasize pre-departure orientation and fostering equitable partnerships between sending and receiving institutions.

## Introduction

Globalization has led to an expanding sphere of influence for high-income country (HIC) academic institutions.^1^^,^^2^ For medical students at these universities, global health experiences are increasingly popular, with an estimated 27% of the United States and 40% of British medical students participating in international medical electives (IMEs) before graduation.^3^^,^^4^ In response, medical schools are providing students with a variety of global learning opportunities, most often in the form of IMEs. These electives in low and middle-income countries (LMICs) are usually month-long rotations in the final year of medical school. However, there is a wide variation in the coursework and preparation involved.^5^^,^^6^ Visiting students are generally supervised by HIC or local physicians. Students may act as observers or be more involved in the medical care of patients.^7^

There is both student and university interest in producing physicians who can work with culturally diverse patients and better understand the global disease burden.^6^ Educational benefits of IMEs include increased tropical disease knowledge and better appreciation of cross-cultural issues in healthcare.^8^^,^^9^^,^^10^^,^^11^ Students who participate in IMEs are also more likely to practice in underserved communities and enter primary care specialties.^9^^,^^12^ For HIC universities, benefits of hosting IMEs include attracting more medical students to their program and increased global recognition.^9^ Furthermore, many host institutions in LMICs benefit financially from sponsoring IMEs through program fees. There is also evidence suggesting that these hospitals gain international recognition and future assistance from these doctors in training.^13^^,^^14^^,^^15^

Although past studies have demonstrated the benefits of IMEs, there is concern whether students are adequately prepared for the clinical and ethical challenges they will face in host countries.^1^ Challenges encountered by visiting students include ambiguous scope of practice, unclear aims of the experience, inadequate supervision, lack of pre-departure training and potential personal safety risks.^16^^,^^17^^,^^18^^,^^19^ These short term engagements may also represent a form of “voluntourism,” as the majority of program benefits are for the sending institution’s students rather than the receiving community.^1^^,^^20^ Visiting students may overburden already resource-limited hospitals by requiring physicians’ time to orient, teach and translate.^21^^,^^22^ Students may make patients uncomfortable, be culturally insensitive, or show disregard for local clinical practice.^23^^,^^24^^,^^25^ Additionally, visiting students have reportedly caused patient harm by practicing beyond their capabilities without licensing or accountability.^15^^,^^16^

With so many potential consequences of the IME for local patients, host physicians, and visiting students, the overall goal of this study is to better understand the experiences of the IME from these varied perspectives. In doing so, the primary objective of this study is to use interviews to elicit the views of Nepali patients and physicians who interact with visiting medical students. We secondarily aim to measure the effects of the IME on visiting medical students’ knowledge of Nepal and clinical competencies using pre- and post-elective surveys.

## Methods

### Study design and participants

Two separate semi-structured interview guides for patients and physicians were developed based on a review of existing literature on IMEs in addition to Nepali faculty input. The finalized interview guides for patients and physicians each included demographic information, in addition to twelve semi-structured questions with probes to understand the benefits and drawbacks of hosting visiting students. Purposive sampling was used to identify 15 physicians who supervise visiting medical students from a variety of medical departments and ranks of academic appointments, including medical officers, instructors, assistant professors, and associate professors. Purposive sampling was also used to identify 15 patients who had recently undergone a consult with a visiting medical student across various departments (Table 1).

Surveys were administered to visiting medical students before and after their medical elective. The survey items were derived from the authors’ experiences working with visiting medical students, as well as previous studies with surveys administered to medical students and residents participating in international experiences.^26^^,^^27^ The pre-elective survey had 23 questions divided into four sections: (1) Demographics, (2) Medical Training, (3) Knowledge of Nepal, and (4) Clinical Competencies. The post-elective survey repeated questions in section (3) Knowledge of Nepal and (4) Clinical Competencies and added a fifth section with 14 questions: (5) End of Elective Feedback. Whenever possible, questions asked participants to respond on a 5-point Likert scale. For the Knowledge of Nepal scale and Clinical Competencies scale, the scoring range was 1 to 5 with a higher score corresponding to a higher level of knowledge or competency. For the End of Elective Feedback scale, the scoring range was 1 to 5 with 1 representing “strongly disagree” to 5 representing “strongly agree.” All survey items also included a free-response section. The survey was piloted with four visiting medical students to ensure the clarity of each question.

**Table 1:** Participant demographics, including department where local patients received consults, host physicians practiced, and visiting students completed their medical elective

Variable		Local patients (n=15)	Host physicians (n=15)	Visiting students (n=56)
Age		Mean (SD)	Mean (SD)	Mean (SD)
		45 (12)	37 (9)	25 (2)
Gender		n (%)	n (%)	n (%)
	Male	9 (60)	10 (67)	22 (40)
	Female	6 (40)	5 (33)	34 (60)
Country of origin				
	Nepal	15 (100)	15 (100)	0
	USA	0	0	17 (30)
	Germany	0	0	16 (29)
	Austria	0	0	9 (16)
	Other European country	0	0	14 (25)
Department				
	Internal medicine	10 (66)	3 (20)	15 (27)
	OBGYN	2 (13)	0	4 (7)
	General surgery	0	3 (20)	8 (14)
	Orthopedic Surgery	3 (23)	2 (13)	4 (7)
	Pediatrics	0	3 (20)	12 (21)
	Emergency Medicine	0	0	7 (13)
	Dermatology	0	2 (13)	4 (7)
	Ophthalmology	0	2 (13)	2 (4)

### Setting

This study took place in Nepal at the non-profit and non-governmental Dhulikhel Hospital, the flagship hospital for Kathmandu University School of Medical Sciences (KUSMS). The hospital attracts over two hundred elective students each year from various HICs. Visiting students request elective placement either through their university partnership or by finding the program online. Each student is assigned to a hospital department, where they are supervised by the attending physicians of that department. Their main activities include observing patient consultations, ward rounds, assisting procedures or operations, and attending conferences. Physicians speak English fluently while most patients speak Nepali language only. At the time of the study, participation in any elective less than six weeks was 200 USD.

**Table 2:** Visiting students’ scores for knowledge of Nepal before and after their medical elective

Survey Item	Pre-elective Mean (SD)	Post-elective Mean (SD)	T score	df	p-value
Knowledge of Nepali language	1.2 (0.4)	1.8 (0.6)	-4.80	55	<0.001
Knowledge of Nepal's culture	2.3 (0.7)	3.0 (0.5)	-5.26	55	<0.001
Knowledge of Nepal's politics	1.8 (0.6)	2.4 (0.7)	-5.07	55	<0.001
Knowledge of Nepal's health system	1.9 (0.6)	3.2 (0.6)	-10.22	55	<0.001
Knowledge of Nepal's epidemiology	1.9 (0.8)	2.9 (0.6)	-7.76	55	<0.001

### Data collection

The study was reviewed in full and approved by the Dhulikhel Hospital Institutional Review Committee (IRC). Written consent was obtained from all students and physicians, and verbal consent was obtained from patients prior to study participation. Author DM conducted 30 to 60 minute digitally recorded, semi-structured interviews in English with supervising physicians regarding IMEs. A Nepali research assistant conducted interviews with patients regarding visiting medical students in the Nepali language. Interviews with patients lasted from 10 to 30 minutes. All interviews were subsequently transcribed in English. Authors SS and DM administered a pre-elective paper survey to all visiting students on the first day of their medical elective during the orientation process, and then post-elective paper surveys on their last day of the elective. Surveys took an average of 15 minutes to complete. Survey results were manually uploaded into Microsoft Excel and cross-checked for accuracy. All 56 students who completed a clinical rotation at Dhulikhel Hospital between Jan 1 and June 7, 2016 were approached to participate in the study. The visiting medical students came from sixteen medical schools across six European countries and the United States. Students stayed an average of 3 to 4 weeks, with the majority of students rotating through the Pediatrics, General Surgery, Emergency, or Internal Medicine departments (Table 1).

### Data analysis

Interview transcripts and free responses to survey questions were transcribed verbatim, translated into English when necessary, and uploaded into NVivo (Version 11, 2016) for qualitative analysis. A conventional content analysis approach was used in order to extract major themes during the first round of transcript analysis.^28^ Emerging themes and coding structures were discussed by the authors, and then a finalized coding scheme was applied to the transcripts. These themes were grouped and reported with descriptive analysis and representative quotes. Descriptive statistical analysis was performed in Microsoft Excel to understand the demographic characteristics of students, physicians, and patients. For Likert-scale survey questions, data were entered into SPSS (Version 23, 2015). Means and standard deviations (SDs) are presented for scale data, in addition to p-values for scales that were administered both before and after the medical elective. Paired t-tests with p<.05 were used to determine statistical significance. The independent variable was pre- versus post- medical elective, while the dependent variables were knowledge of Nepal and clinical competencies.

## Results

### Patient and physician positive perceptions of the IME

Enhanced reputation of hospital in the community: Multiple patients and physicians believed that Dhulikhel Hospital’s reputation in the community was enhanced by the presence of visiting students. One patient stated that “When foreign doctors come to treat patients, then the patients will be cured…so yes, more patients will come here.” Physicians also thought that their patients were eager to have visiting students, with one physician reporting: “Our patients are always happy to see white faces and will trust our department more even though the foreigners are just students.”

Increased international collaboration: In addition to improved local reputation, many physicians also believed the IME improved the international recognition of the hospital. This relationship was seen as mutually beneficial for both host physicians and visiting students, with one physician commenting: “It is good to have the students come here, because then we can make relationships with foreign universities and even go there.” These international partnerships allowed for professional development opportunities for Nepali physicians, with six of the physicians interviewed attending conferences or completing advanced fellowships in the USA or Europe.

Financial benefits for the hospital: A few patients and physicians mentioned the financial benefits of hosting visiting students, both in terms of directly assisting the hospital, and tourism in Nepal more broadly. Visiting students were required to pay a 200 USD program fee, which physicians reported was helpful to the hospital generally, but did not necessarily go to specific departments. A patient stated, “Having the students come is a good thing… They will spend some money here and may go for tours around Nepal.” One physician reported that, “sometimes students bring some donations, like blankets, heaters and other equipment.”

Knowledge, culture, and language exchange: Patients and physicians reported that one of the strengths of the IME was gaining a better understanding of medical practice in HICs, including new advances in diagnostics and treatment. Patients specifically reported that visiting students may bring knowledge of novel treatments, technologies, and sanitation. Some patients also believed that visiting students could help in the hospital’s development, so that “Dhulikhel Hospital will become more like foreign hospitals.” Most supervising physicians regarded the visiting student elective as a chance to practice their English and learn about how medicine training and practice occur in HICs. Many physicians echoed the sentiment that: “It’s always good to have people from different cultural and medical backgrounds because we are able to learn from each other.”

Patient Satisfaction: All of the 15 patients interviewed felt comfortable interacting with visiting medical students and felt positive about having visiting students working at Dhulikhel Hospital. As one patient stated, “half of curing disease is making the patients happy, so the foreigner’s presence has a positive impact on our health.” The doctors also felt that their patients liked having visiting students observing. One physician believed that the patients “will feel better about coming to our hospital because they think the foreigners will offer better care.”

**Table 3:** Clinical competencies scores for visiting medical students before and after their medical elective

Survey Item	Pre-elective Mean (SD)	Post-elective Mean (SD)	T score	df	p-value
Ability to communicate with patients from different cultural and socioeconomic backgrounds	3.3 (0.8)	3.2 (1.0)	0.37	55	0.79
Ability to communicate with health professionals from different cultural and socioeconomic backgrounds	3.3 (0.7)	3.6 (0.7)	-3.11	55	0.003
Ability to quickly adapt to a new healthcare setting	3.4 (0.8)	3.5 (0.8)	-0.68	55	0.50
Ability to practice in resource-constrained environments	2.4 (0.9)	2.8 (0.9)	-2.42	55	0.02
Ability to conduct patient histories	4.0 (0.7)	3.9 (1.0)	0.84	55	0.40
Ability to perform a physical exam	3.9 (0.6)	3.9 (0.7)	0.23	55	0.82
Ability to reach a patient diagnosis	3.5 (0.7)	3.4 (0.8)	1.52	55	0.14
Ability to recommend appropriate medications	3.0 (0.8)	2.9 (0.8)	0.94	55	0.35

### Patient and physician negative perceptions of the IME

Time burden: For physicians, the major drawback of the program was a lack of compensation for extra time spent in assisting with translation, orientation, and teaching for visiting students. Although visiting students paid a fee for the elective, individual physicians and departments were not directly compensated. Some individual faculty and departments faced a much larger teaching burden than others, especially those in internal medicine, pediatrics, and general surgery. During one month of the study, the pediatrics department hosted six visiting students at once, leading one pediatrician to comment, “we don’t mind having [visiting students], but sometimes if many come at once, it can be quite busy to balance teaching them with our other duties.”

Visiting students detract from local student learning: When probed for drawbacks of hosting visiting students, one patient commented that “extra students can make the hospital feel crowded.” Another patient mentioned that visiting students may interfere with Nepali student learning by “taking away opportunities from Nepalis.” Two physicians also commented that the visiting students may take away Nepali students’ opportunities in the hospital. One surgeon stated: “I want them [visiting students] to help do the operations, and scrub-in with me, but some Nepali students may be upset that their opportunity has been taken by the international student.”

Language barrier: For patients, one of the biggest drawbacks was their inability to communicate in English with the visiting students. None of the patients interviewed spoke English with proficiency but were often communicated in English by visiting students. One patient said: “I could not completely understand the foreigner due to the language barrier, but she gave me good advice to do a chest X-Ray and blood test.” Another patient said that he was unsure about what the student and physician were talking about in English during his consult, though overall he still felt positive about the care he received.

Excess vacation: Physicians were frustrated that most visiting students only stayed for a few weeks, with one stating, “We don't know what they're interested in, and some don't have much training. They just come for a very short period of time.” Three physicians in particular perceived the visiting students as coming to Nepal for vacation and working minimal hours at the hospital. Students engaged in tourist activities such as Himalayan treks without notice, making it difficult for departments to accommodate them. One physician lamented that “Sometimes it seems like their main motive is just to roam around the countryside, and at that time we feel really bad.”

Patient misconceptions: Although none of the patients interviewed felt mistrustful of the students, many of the patients had misconceptions about whether the visiting students were students or doctors. For example, one patient said, “The foreigners may be doctors, or they may be students, I cannot tell.”

Critique of medical practice: One physician complained about visiting students’ criticism of how medicine was practiced at the hospital. He shared that, “Some students will blame us for prescribing too many antibiotics. They are completely unaware of the situation.”

### Visiting student survey findings

Visiting students found the elective through their university (46%), online (44%), or through friends (9%). Only 4% of students received pre-departure orientation for their elective and 34% of students had prior medical experience in a resource-limited setting. Around 70% of students financed their travel with a university scholarship, which covered from 700 to 1,000 USD. Visiting students’ main objectives included experiencing a new healthcare system (86%), engaging in Nepali tourism (86%) and learning about diseases endemic to Nepal (72%).

In the self-reported knowledge section of the survey, students demonstrated a significant increase in knowledge of Nepali language, Nepal’s culture, Nepal’s politics, Nepal’s health system, and Nepal’s epidemiology after the medical elective (Table 2). In the clinical competencies section of the survey, there was a self-reported increase in students’ ability to communicate with health professionals from different cultural backgrounds and ability to practice in resource-constrained environments; however, there was no significant increase in self-reported ability to conduct patient histories, perform a physical exam, reach a diagnosis or prescribe appropriate medications (Table 3).

In the post-elective feedback part of the survey, students were overall satisfied with the diversity of medical cases and teaching by the faculty. However, students did not feel that the rotation provided appropriate skills training and many felt there was a lack of supervision. Most students conveyed that the benefits of the rotation included that it increased their appreciation for the impact of health on culture and that it was personally rewarding. However, many students did not feel adequately connected with the hospital or broader community and overall did not believe they were making a difference in patients’ lives (Table 4).

**Table 4:** Visiting students’ end-of-elective feedback responses on a 5 point Likert scale, where 5 is strongly agree and 1 is strongly disagree

Preface	Survey Item	Mean (SD)
My elective experience in Nepal…	Increased my appreciation for the impact of culture on health	4.1 (0.6)
	Provided an appropriate diversity of cases	3.9 (0.8)
	Was personally rewarding	3.7 (1.0)
	Provided a good teaching relationship between students and faculty	3.7 (1.1)
	Taught appropriate medical knowledge	3.5 (0.9)
	Connected me with the hospital community	3.5 (1.0)
	Provided an appropriate level of supervision	3.3 (1.1)
	Increased my sense of social responsibility	3.2 (1.2)
	Provided an appropriate clinical training experience	3.0 (1.1)
	Can be described as “life-changing"	2.7 (0.9)
	Was valued by the community	2.6 (1.0)
	Taught appropriate clinical skills	2.3 (1.1)
	Made a difference in patients' lives	2.2 (0.9)
	Affected my choice of medical specialty	1.9 (1.2)

In written feedback, visiting students most appreciated experiencing a culture and healthcare system different from their own. A few participants felt they wanted to return to Nepal to engage in further clinical, service or research opportunities, with one student writing: “I've been fascinated by the country. The language, people, and different diseases have made me think I may be interested in gynecology. I want to come back here as a doctor and be able to make changes.” However, other students realized the challenges that working abroad can pose, and decided that focusing on disparities within their own countries would be more fulfilling.

Students often felt “lost” during their electives, especially in departments where students were not assigned to a particular supervisor. Although all students were able to observe patient consults and procedures, most wished they could have played a more active role on the team. In the words of one respondent, “We learned a lot by watching. The supervisors always answered all our questions, but we could not do much practical training.” This mirrored interviews with physicians, where the majority of physicians said that the students were only allowed to observe. A minority of students also had trouble adapting to the hospital environment in Nepal, which had different follow-up, privacy, sanitation, and prescribing procedures than they were accustomed to.

## Discussion

Since the 1970s, the IME has been regarded by many as a highlight of the medical school curriculum.^8^^,^^19^^,^^29^ Recently, there has been a more substantial emphasis on how host physicians and patients may receive visiting students. Our study revealed that patients generally believed that their medical

care was enhanced by the presence of visiting students. As noted in previous studies, patients felt that student involvement helped directly with patient care and would positively impact their community.^15^^,^^23^ Part of this universally positive reaction to HIC students may be related to the misconception that students were physicians. In the future, there should be more effort to inform patients about the role of visiting students to avoid this confusion.

Similar to patients, physicians generally viewed visiting students positively. Students provided glimpses into HIC medical practice and afforded an opportunity to practice speaking English. Another benefit to physicians was increased prestige of their hospital in the local community and internationally. As seen in other studies, some physicians had research and other academic ties to sending universities, which they believed greatly enhanced their own professional development.^5^^,^^14^^,^^21^^,^^30^^,^^31^ However, physicians also reported that visiting students strained already limited resources. Although all students paid a fee to the hospital, physicians were not directly compensated for teaching students. Further, some departments and individual physicians had much greater teaching and orienting burden than others.^5^^,^^32^ In our study, visiting students sometimes competed with Nepali medical students for teaching time from physicians, a finding which has not been well described. For physicians, the biggest time commitment was translating patients’ histories from Nepali to English. Enhanced pre-departure language training or linkage of visiting students with Nepali trainees may have alleviated some of this burden. Fair compensation for physicians and adequate allocation of time for both local and visiting students also needs to be addressed.

Host physicians were often disappointed by visiting students’ failure to fully engage in medical activities and fulfill their commitments of the program, which was also noted in a prior study.^21^ The one month length of the rotation did not give students enough time to appropriately integrate into their team, leaving both physicians and students feeling unengaged.^33^ Similar to results of another study, certain students criticized medical procedures and conditions specific to working with resource limitations.5 Sending universities and host institutions should work together to develop more stringent IME guidelines to ensure students act professionally and responsibly while abroad.

The benefits visiting students receive have been well documented in the literature, including exposure to social determinants of health, understanding the impact of culture on healthcare, and learning tropical disease presentation.^9^ Many of the challenges experienced by visiting students reflected a mismatch in expectations and realities of the medical student elective, generally revealing a lack of pre-departure information and oversight from supervisors at sending and local institutions. In particular, visiting students scored low in self-assessed knowledge of the social, political, cultural and linguistic context of Nepal. For many, the highlight of the experience occurred outside of the clinic while touring Nepal’s countryside.^30^ Past studies found that students were “practicing on the poor,” causing dependency on foreign medical care.^15^^,^^16^^,^^24^ However, in this study many students felt they did not practice what they considered basic skills taught at their home institution, such as physical examination and minor procedures. For the students in this study, the elective represented more of an observational than clinical experience, which may be difficult to avoid without appropriate pre-departure training.

Many studies have emphasized the need for pre-departure training to improve benefits for both students and hosts. However, pre-departure training is still not the norm, with one study of Canadian medical schools finding that 44% of program directors allow IMEs to occur without clear faculty oversite or input.^34^ Specifically, this training should include broad issues in global health, especially potential ethical dilemmas that students might encounter, as well as country-specific medical, cultural, language and health systems training.^12^^,^^13^^,^^35^ This comprehensive orientation may reduce the likelihood of students feeling disconnected during the elective by providing them with necessary information to better engage with Nepali physicians and patients.

Too often, electives benefit visiting students without enhancing the health of local patients. These programs have been described as a “one-way process” that unilaterally benefits students from HICs.^36^ Increasingly, medical schools have been creating bidirectional partnerships at hospitals in LMICs in order to establish long term commitments. The students in our study came from sixteen medical schools across seven countries. The majority of these students found the IME through online elective networks instead of through a university-sponsored programs. However, three of the universities who sent medical students had a partnership with the hospital in Nepal. Host physicians felt these partnerships allowed for better communication and feedback between sending and hosting university, as well as the opportunity for Nepali physicians to receive further training at these universities. Although a bi-directional model is likely better than the parachute model adopted by many HIC medical schools, these bidirectional partnerships may have unintended consequences as well.^37^^,^^38^ One author dubs these partnerships as “a 21st century scramble for Africa,” a title that increasingly refers to linkages between HIC and LMIC universities.^1^

Our study has many limitations. This study was conducted with a small sample size at one academic hospital in Nepal, which constrains how generalizable the findings may be to other settings. Purposive sampling of physicians and patients was used, which may have restricted the diversity of responses. The interviewer for physicians came from a HIC, which may have biased the way in which questions were answered. The survey was developed by the authors and was not derived from previously validated work, limiting the reliability of the findings. Furthermore, a Likert scale was used, which may result in central tendency and acquiescence biases.

## Conclusions

Overall, this study demonstrated that the IME in Nepal improves European and American medical students’ cultural competence and ability to work in a resource-limited setting, while also benefiting patient care. However, these elective opportunities must be organized with clearer learning objectives and ethical principles in mind to be most beneficial to the medical students themselves, while also taking into consideration potential harms to hosting physicians and local patients. Pre-departure orientation is greatly needed by sending HIC medical schools in order to improve students’ knowledge of the destination country beforehand. Furthermore, the partnership model between HIC medical schools and LMIC hospitals may offer a more equitable exchange of knowledge and resources across institutions. Additional studies capturing more diverse sets of views, especially from various local stakeholders and communities, are needed.

### Acknowledgments

The authors would like to acknowledge Suraj Makaju for assistance with conducting interviews and translation. This project was funded by the US Student Fulbright Research Grant, Nepal.

### Conflict of Interest

The authors declare that they have no conflict of interest.
